# Enhanced Transmembrane Delivery of Chemotherapeutic Agent Doxorubicin by Carbon Nanotubes Under Plasma Synergy: Molecular Dynamics Insights

**DOI:** 10.3390/biom16050679

**Published:** 2026-05-03

**Authors:** Roujia Lin, Tong Zhao, Yanxiong Niu, Xiaolong Wang, Ying Sun, Yuantao Zhang

**Affiliations:** School of Electrical Engineering, Shandong University, Jinan 250061, China; linroujia@mail.sdu.edu.cn (R.L.); niuyx@mail.sdu.edu.cn (Y.N.); wangxiaolong@sdu.edu.cn (X.W.); ys2018@sdu.edu.cn (Y.S.); ytzhang@sdu.edu.cn (Y.Z.)

**Keywords:** cold atmospheric plasma, carbon nanotube, doxorubicin, molecular dynamics simulation, synergistic anticancer effect

## Abstract

Doxorubicin (DOX) is widely used in clinical chemotherapy, but its susceptibility to oxidation during the combined treatment with cold atmospheric plasma (CAP) raises concerns regarding its therapeutic efficacy. To improve drug stability and targeted delivery efficiency, this study employed classical molecular dynamics simulations to systematically investigate the mechanisms by which CAP-generated active particles and electric fields influence DOX encapsulation by carbon nanotubes (CNTs) and their transmembrane transport. Within a specific range of active particle concentrations, DOX aggregation is suppressed, enabling its spontaneous entry into CNTs for encapsulation. The CAP-induced electric field further promotes the directional migration of DOX, and once a threshold field strength is reached, the encapsulation efficiency is significantly enhanced. Moreover, an appropriate concentration of active particles can lower this threshold, enabling high encapsulation efficiency at electric field strengths as low as 0.3 V/nm. The introduction of CNTs can reduce the exposure of DOX to active particles, thereby effectively protecting it from CAP-induced oxidation. Regarding transmembrane transport, CAP-induced lipid oxidation decreases membrane structural stability, facilitating the intracellular internalization of CNTs and promoting the release of DOX within target cells. Furthermore, under the combined effects of oxidation and electric fields, the pulling force required for CNT transmembrane transport further decreases, the size of transmembrane pores increases, and the transmembrane delivery of DOX is enhanced. These results demonstrate that, under plasma synergy, CNTs exhibit significant potential in enhancing the targeted delivery of chemotherapeutic agents. This work provides important theoretical support for the application of plasma in targeted cancer therapy and offers new insights for the design of precision cancer treatment strategies.

## 1. Introduction

Cancer poses a serious threat to human life and health, and its incidence and lethality are increasing globally every year [1]. Despite progress in cancer treatment using antitumor drugs, these treatments can cause serious adverse effects when applied clinically [2]. Moreover, cancer cells can become resistant to chemotherapy drugs during long-term treatment, thus limiting the effectiveness of cancer treatment [3]. Cold atmospheric plasma (CAP) sources have great potential for synergizing with anticancer drugs in the treatment of cancer and are the latest application of plasma biomedicine, which is currently receiving considerable attention [4]. CAP is an ionized gas composed of ions, electrons, free radicals, reactive neutral substances, etc., and is rich in reactive oxygen species (ROS) and reactive nitrogen species, which can cause irreversible damage to critical parts of cancer cells, such as DNA, RNA, and mitochondria, leading to oxidative stress and ultimately to apoptosis [5]. Compared with either plasma alone or drugs alone, plasma in combination with anticancer drugs has been extensively studied for the treatment of cancer, restoring the sensitivity of cancer cells to drugs and enhancing therapeutic effects, and it has shown great potential for the treatment of gliomas and pancreatic cancer [6,7].

Doxorubicin (DOX) is a widely used anticancer drug that breaks the DNA of cancer cells and increases the amount of ROS in cancer cells [8,9]. Its synergistic effect with plasma has been extensively studied, with higher concentrations of ROS observed in cancer cells after synergistic treatment than in cells treated with plasma or DOX alone [10,11]. Studies have shown that the synergistic treatment of plasma and DOX reduces the lymphosarcoma tumor volume by 2.5-fold, decreases the probability of prognostic recurrence [12], and greatly enhances the tumor toxicity of DOX at very low concentrations [13]. However, experimental studies have shown that non-thermal/cold plasma can induce oxidative degradation of pharmaceutical compounds, and such plasma-induced chemical modification may reduce their biological activity or functional performance [14,15,16]. In addition, forced degradation studies have demonstrated that DOX is sensitive to oxidative conditions and can form multiple oxidative degradation products [17]. Therefore, under CAP-assisted conditions, protecting DOX from possible oxidation is an important issue for maintaining its therapeutic effectiveness. Moreover, the systemic effects of DOX can also cause serious adverse effects, i.e., not only irreversible myocardial damage but also an increased risk of leukemia [18]. In order to enhance the synergistic therapeutic efficacy of DOX in combination with CAP, this study explores the use of carbon nanotubes (CNTs) as a co-delivery vehicle for DOX. Single-walled carbon nanotubes (SWCNTs) are hollow cylinders made of graphite flakes with diameters ranging from approximately 0.5 nm to 5 nm. They have an excellent binding capacity with DOX, while their hydrophobic property can selectively inhibit the entry of hydrophilic oxidants into the interior of CNTs [19,20,21,22], and the large conjugated double bond arrays make them excellent electron donor-acceptors, so that oxidants will preferentially oxidize the CNTs rather than the drug [23]. Meanwhile, CNTs tend to accumulate near cancer cells due to the Enhanced Permeability and Retention effect [24]. Thus, CNTs are capable of preventing DOX from being oxidized by CAP during CAP-induced cancer cell apoptosis. At the same time, they enhance the transmembrane delivery of DOX, thereby potentiating the synergistic anticancer efficacy of the CAP–DOX combination.

CNTs possess high drug-loading capacity, excellent antioxidant properties and the ability to penetrate cells, as well as excellent electrical and mechanical properties that can enhance the applied electric field through their high aspect ratio [25]. Moreover, some plasma sources are also capable of generating strong electric fields ranging from a few to 100 kV/cm. These strong electric fields are capable of inducing the formation of pores in the phospholipid bilayer, which enhances cellular electro-permeability [26], resulting in the electroporation and apoptosis of cancer cells. The oxidative effect of active particles acting in conjunction with an electric field may help CNTs encapsulate and protect drugs while facilitating their translocation across cell membranes at lower field strengths [27], thereby promoting drug delivery. The synergistic effects of CAP and nanodrug delivery systems on apoptotic cancer cells have shown potential. For example, Zhu et al. reported that plasma treatment improved the cellular internalization of nanoparticles [28]. Kim’s study revealed that the synergistic effects of plasma with antibody-conjugated gold nanoparticles dramatically increased the mortality rate of melanoma cells [29]. In Cheng’s investigation, plasma treatment accelerated nanoparticle endocytosis [30]. Finally, previous studies have also reported the increased adsorption capacity of plasma-treated CNTs [31]. Compared with gold nanoparticles, CNTs are more capable of encapsulating drugs and preventing them from being oxidized, and on the basis of these considerations, the synergistic effect of plasma and CNTs for the delivery of DOX holds considerable promise. Although researchers have conducted many experimental studies on the synergistic effects of nanodelivery systems and plasma, the detailed mechanism by which plasma assists CNTs in delivering drugs for cancer treatment at the molecular level is not yet known. Furthermore, it is difficult to optimize the dosage to obtain the optimal strategy for treating apoptotic cancer cells.

In recent years, molecular dynamics simulations have gradually developed into a third means of scientific research, following theoretical analysis and experimental observation. In the field of plasma medicine, the most fundamental and intuitive role of molecular dynamics simulations is to explore the interaction processes between active particles and biological structures, aiming at solving microscopic-level questions that are difficult to answer experimentally [32]. Molecular dynamics simulation methods are promising tools for computer design for drug delivery [33] and have been widely used to probe the intrinsic mechanisms of nanodelivery systems [34,35,36]. These methods are capable of generating important insights into the process by which CNTs encapsulate DOX and cross the cell membrane in the plasma environment.

**Figure 1:**
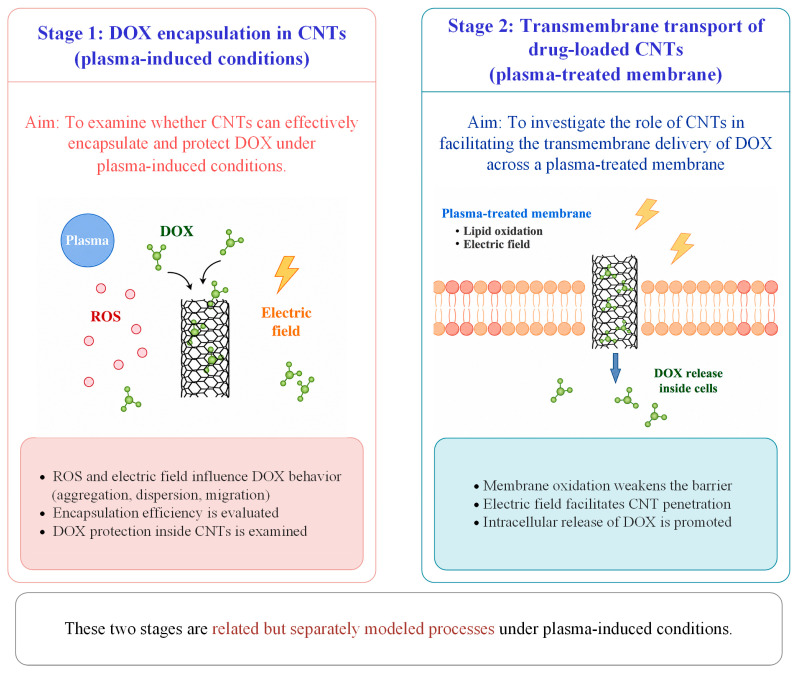
Schematic illustration of the two-stage modeling strategy. In Stage 1, the arrows indicate the tendency of DOX molecules to enter the CNT. In Stage 2, the arrow indicates the transmembrane transport of the drug-loaded CNT across the plasma-treated membrane.

In this work, the mechanisms by which CAP affects the encapsulation of DOX by CNTs and its passage across cell membranes are examined using classical molecular dynamics simulations. It should be noted that this work adopts a simplified two-stage modeling strategy. A schematic illustration of the two-stage modeling strategy is provided in Figure 1. In the first stage, plasma-induced conditions are considered to investigate whether CNTs can still effectively encapsulate and protect DOX. In the second stage, the role of CNTs in facilitating the transmembrane delivery of DOX across a plasma-treated membrane is studied. This approach enables a focused analysis of the key mechanisms of CNT-assisted drug delivery without explicitly reproducing the full temporal sequence of plasma treatment and drug administration. First, the effects of increased ROS concentration and electric field strength on the ability of CNTs to encapsulate and protect DOX were investigated under plasma ROS and electric field conditions; Second, an oxidized phospholipid bilayer model (POPC-ALD) was used to study the effects of plasma oxidation on the transmembrane transport mechanism of CNTs. Subsequently, an electric field is applied to the POPC bilayer model to probe CNTs across membranes with different electric field strengths, and the influence of oxidative effects synergized with electric fields on CNTs across the membrane is also considered. The results show that, in the presence of active particles together with an electric field, the encapsulation and protective capabilities of CNTs for DOX are further enhanced, effectively preventing the oxidation of DOX during synergistic interaction with plasma and inhibiting the aggregation of DOX into clusters, thereby increasing its potential in cancer therapy; the synergistic effect of oxidation effect with the electric field of CAP enhances the intracellular internalization of the CNTs, promotes DOX transmembrane delivery, and strengthens the penetration of the active particles through the membrane pores, thus facilitating the destruction of key intracellular components by the active particles in concert with DOX. This paper reveals the microscopic mechanism by which CNTs encapsulate DOX and cross the cell membrane under the multiple effects of CAP. This discovery not only provides new design ideas for drug delivery systems to synergize CAP for cancer treatment but also provides an innovative theoretical basis for precision-targeted cancer therapy.

## 2. Materials and Methods

### 2.1. Modeling of the Adsorption and Encapsulation of DOX in Carbon Nanotubes

**Figure 2:**
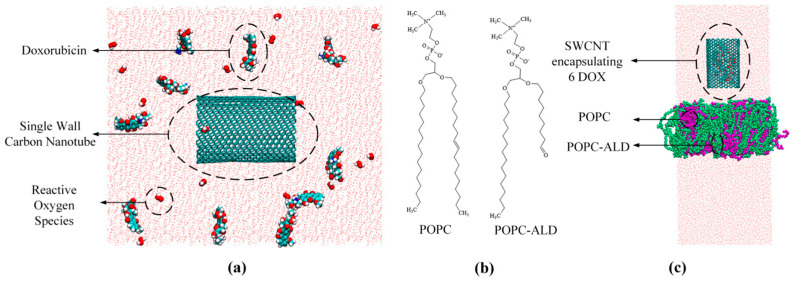
(**a**) Model of a CNT encapsulating DOX; (**b**) Molecular structure of POPC and POPC-ALD; (**c**) Model of a drug-loaded CNT crossing an oxidized POPC membrane. Water molecules are shown in pink, the CNT is shown in dark green, DOX molecules are represented by red, cyan, and blue atoms, POPC is shown in purple, POPC-ALD is shown in light green, and ROS particles are represented by red and white atoms.

In this study, a two-stage modeling strategy is adopted, in which DOX encapsulation in CNTs and the transmembrane transport of drug-loaded CNTs are treated as two related but separately modeled processes under plasma-induced conditions, enabling a focused analysis of the underlying mechanisms. Studies have shown that the toxicity of active H_2_O_2_ particles plays an important role when combined with DOX in the treatment of cancer. When combined with DOX, the concentration of H_2_O_2_ in cancer cells increases significantly, making it one of the main active particles in both the intracellular and extracellular environments [37]. In addition, OH• and OOH• are key reactive oxygen species with strong oxidative activity. To more comprehensively characterize the ROS environment and more realistically simulate plasma-induced oxidative conditions, OH•, OOH• and H_2_O_2_ were selected as the main particles. These particles were used to investigate how ROS modulate the molecular state and dynamic behavior of DOX, thereby indirectly influencing its encapsulation and protection by CNTs in an ROS environment generated following plasma treatment. A 10 × 10 × 12 nm^3^ box was set up using PACKMOL program [38], and a 4 nm long SWCNT was placed at the center of the system along the *z*-axis. The SWCNT had chiral indices of (20,20), corresponding to an armchair SWCNT, which has been reported to be favorable for DOX encapsulation [21]. It has been previously reported that DOX conjugated with antibodies has the strongest therapeutic effect on cancer at an antibody/DOX ratio of 1:8–10 [39,40]. This ratio was used here only as a general reference to select a reasonable number of DOX molecules for the simulation system. Accordingly, 10 DOX molecules were randomly placed around the SWCNT model using PACKMOL to ensure a reasonable loading level while avoiding excessive aggregation of DOX molecules. In addition, 12, 18, and 24 ROS particles were added to the box to investigate the molecular mechanism of DOX encapsulation and protection by CNTs at different ROS concentrations, as shown in Figure 2a. These ROS concentration levels were adopted from a previous study [41]. For each ROS concentration, equal numbers of OH•, OOH• and H_2_O_2_ were included; that is, in the systems containing 12, 18, and 24 ROS particles, the number of each ROS was 4, 6, and 8, respectively. This simplified setup was used to provide a more representative description of the plasma-induced ROS environment. Since a high length-to-diameter ratio of CNTs can amplify the electric field effect of CAP, the effect of the co-presence of electric field and active particles on the encapsulation and protection of DOX in CNTs was investigated. The TIP3P water model, a widely used three-site rigid water model in molecular dynamics simulations, was employed to solubilize all simulated systems. And NaCl was added at a concentration of 150 mM. All simulations of the adsorption and encapsulation of DOX in CNT were performed under the NVT ensemble. In this ensemble, the number of particles, the volume, and the temperature are kept constant. This setup allows a more focused analysis of the intermolecular interactions and the adsorption and encapsulation behavior in a fixed-volume system, where density fluctuations are negligible and do not significantly affect the adsorption and encapsulation process. Maintaining a constant volume ensures system stability and allows for a more accurate analysis of the interaction mechanisms among DOX, CNTs, and ROS. Prior to the production simulation runs, energy minimization was carried out using the conjugate gradient algorithm to eliminate unfavorable contacts in the system. Electrostatic interactions were treated using the particle mesh Ewald method. A V-rescale thermostat was used to keep the temperature (310 K) constant, with a cutoff radius of 1.4 nm and a simulation time step of 2 fs for the method considered. The LINCS algorithm was used to maintain the rigidity of the molecular bonds, with periodic boundary conditions applied in three dimensions. All simulations in this study were performed using the GROMACS software package (version 2023.2) with the GROMOS54A7 force field. The molecular structure of DOX was obtained from DrugBank (https://go.drugbank.com/, accessed on 15 October 2025) [42], the topology of DOX was obtained from the Automated Topology Builder (https://atb.uq.edu.au/, accessed on 15 October 2025) [43], and the molecules were visualized using the Visual Molecular Dynamics package (version 1.9.3) [44]. To avoid randomness in the simulations, each simulation was repeated 3 times under the same conditions.

### 2.2. Modeling of Carbon Nanotubes Across Cell Membranes Under the Plasma Effect

Since the drug delivery properties of SWCNTs depend on their ability to penetrate cell membranes, the oxidative effect of plasma and the ability of electric fields to assist CNTs in crossing cell membranes were investigated in this study. A phospholipid bilayer of 200 POPC (100 per layer) molecules was constructed, which is a commonly used system size in molecular dynamics simulations of lipid membranes to ensure structural stability and reduce boundary effects [45,46,47]. CNTs encapsulating 6 DOX molecules, corresponding to a representative loading state obtained under plasma-induced conditions, were placed in the water phase approximately 1.5 nm above the bilayer under an electric field of 0.3 V/nm. It was observed that cell membranes were oxidized to produce aldehyde groups in experiments [48]. In addition, Wang’s study showed that the aldehyde oxidation product of POPC had the most significant effect on plasma interaction [49]; therefore, the oxidized membrane lipid structure was constructed with a 40% aldehyde oxidation product (POPC-ALD) and 60% POPC, as shown in Figure 2c. The simulations of transmembrane transport were carried out under the NPT ensemble. In this ensemble, the number of particles, the pressure, and the temperature are kept constant. This is because membrane systems require pressure coupling to maintain the correct lipid accumulation density and membrane tension. The NPT ensemble allows the bilayer to dynamically adjust its area and thickness, which is crucial for accurately capturing membrane deformation, pore formation, and CNT transmembrane transport processes. The initial conformation of the bilayer underwent a 100 ns equilibrium simulation to construct a CNT transmembrane model and then again underwent NVT, NPT equilibrium for steered molecular dynamics simulations. A V-rescale thermostat and a C-rescale barostat were employed to maintain the temperature and pressure at 310 K and 1 bar, respectively. The center of mass of the CNT was attached to the virtual atoms via a virtual spring with a spring constant of 1000 kJ/mol and was pulled at a constant velocity of 0.003 nm/ps; the pulling process was constrained by the same force in the x and y direction. Equations (1) and (2) were used to calculate the force required to pull the SWCNTs:

(1) F=−∇U,

(2) $$U=\frac{1}{2}K(\nu t-\left(\vec{r}-{\vec{r}}_{0}\right)\cdot \vec{n}{)}^{2},$$

where ∇U is the potential energy gradient, K is the spring constant, t is the current time, $\vec{r}$ is the position of the virtual atom, ${\vec{r}}_{0}$ is the initial position of the virtual atom, and ν is the pulling speed.

## 3. Results and Discussion

### 3.1. Adsorption and Encapsulation Mechanism in the Presence of Plasma-Induced ROS

**Figure 3:**
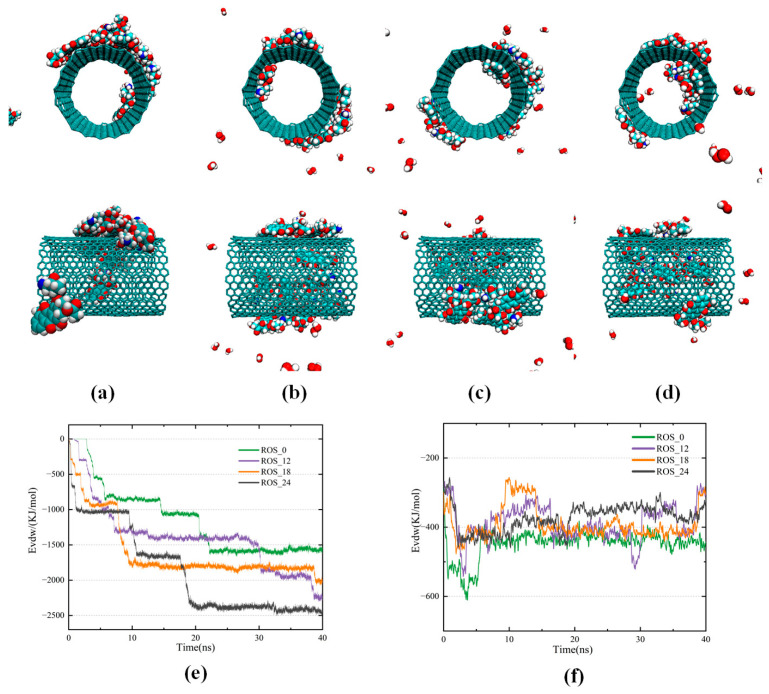
Front and side view snapshots of CNTs combined with DOX for different concentrations of active particles after 40 ns of simulation with (**a**) zero ROS particles; (**b**) 12 ROS particles; (**c**) 18 ROS particles; (**d**) 24 ROS particles; (**e**) Variations in van der Waals energies between CNTs and DOX over the 40 ns simulation under different ROS concentrations; (**f**) Changes in van der Waals energies between DOX molecules during simulation.

In this section, the adsorption and encapsulation behavior of DOX inside and outside CNTs under different ROS conditions is investigated, with particular focus on how CNTs bind and protect DOX from oxidation in a plasma-induced environment at the microscopic level. When no ROS particles are added to the system, one DOX molecule enters the inside of the CNT, and the remainder are attached to the outer wall of the CNT, but some of the DOX accumulates to form clusters that are not close enough to the CNT. When 12 ROS particles are introduced into the DOX-containing environment, interactions between ROS and the hydrophilic groups of DOX partially weaken the π–π stacking interactions between DOX molecules, resulting in a reduction in the van der Waals forces and a decreased tendency to aggregate, and moreover, collisions between ROS particles and DOX enhance the mobility of DOX particles, making it easier for them to move into the CNT interior. Encapsulation of DOX within CNTs reduces its exposure to ROS, thereby protecting it from oxidation. If CNTs are absent, DOX is more prone to interaction with ROS and subsequent oxidation, and this oxidative effect becomes increasingly pronounced with rising ROS concentration; when the number of ROS particles rises to 18, DOX molecules located near the CNT rapidly enter the CNT, reducing their exposure to ROS, whereas those farther from the CNT remain unable to enter independently, instead colliding with neighboring DOX, aggregating into clusters, and finally adhering to the outer wall of the CNT, where they remain susceptible to oxidation by ROS; when the ROS particles count increases to 24, DOX molecules located near the two ends of the CNT are able to enter the inner cavity very quickly, with three of their aromatic rings aligning along the CNT and orienting parallel to the tube wall, while the fourth ring does not tilt toward the CNT, forming a typical face-to-face (F-type) π–π stacking configuration. This stable combination with the CNT effectively protects DOX from oxidation. And at this point, a total of 4 DOX molecules are encapsulated within the CNT, indicating efficient loading. With increasing ROS concentration, DOX more readily enters and stably resides within the nanotube, and the encapsulation and protective capabilities of CNT are enhanced, effectively preventing oxidation of DOX. Since SWCNTs are electrostatically neutral, van der Waals interactions are the main driving force for the encapsulation of DOX. Figure 3e shows that the van der Waals force between DOX and the CNT increases by the addition of active particles to the environment. This indicates that the number of DOX adsorbed onto or encapsulated within the CNTs increased. The van der Waals energy decreases rapidly when the ROS particles count increases to 18, and it can be concluded that the binding of DOX to CNT accelerates and that the encapsulation is essentially complete at around 10 ns, thereby reducing the exposure of DOX to ROS and lowering the likelihood of oxidation. The utilization of 24 ROS particles not only accelerated the process of DOX entering the CNT but also increased the binding energy, which greatly increased the number of DOX entering CNT to 4, indicating that the encapsulation and protective capabilities of CNT were improved. The van der Waals energy between DOX molecules in Figure 3f reflects their aggregation behavior. As the ROS concentration increases, the van der Waals energy gradually increases, and the van der Waals force gradually decreases, which suppresses DOX aggregation, promotes its entry into CNT, and reduces the likelihood of oxidation to DOX. These findings indicate that, under certain ROS concentrations, CNTs can spontaneously adsorb and encapsulate DOX, thereby protecting it from oxidation during the delivery process, improving the efficiency of targeted treatment of cancer cells.

### 3.2. Adsorption and Encapsulation Mechanism in Active Particles and Electric Field Environment

CNTs can enhance the applied electric field by their high aspect ratio, and some CAP sources are also capable of generating strong electric fields ranging from a few to 100 kV/cm, affecting the movement of polar molecules. Therefore, the effect of the CAP-induced electric field on the encapsulation and protection of DOX by CNTs was further investigated at the molecular level. Electric fields of varying intensities (0.1–0.5 V/nm) were introduced into systems containing 18 and 24 ROS particles, and the number of DOX molecules entering the CNTs was monitored. These results were then compared with those from control groups subjected to the electric field in the absence of active particles. This setup was designed to evaluate how the combined effects of ROS and electric fields influence DOX encapsulation and the protective role of CNTs under plasma-induced conditions.

**Figure 4:**
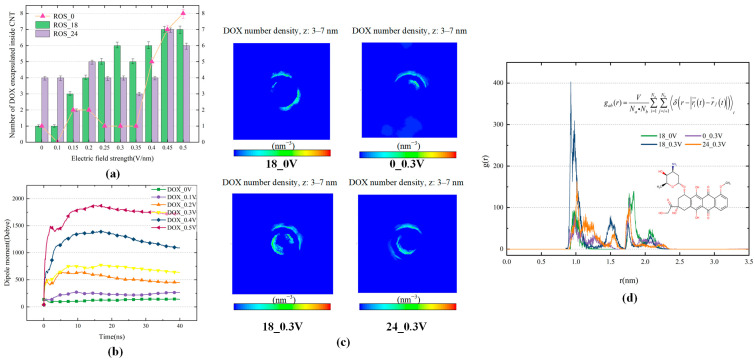
(**a**) Variation in the amount of DOX encapsulated inside CNTs when a 0.1–0.5 V/nm electric field is applied to systems with 18 and 24 ROS particles versus no plasma-generated active particles added; (**b**) Dipole moments of DOX when electric field is applied to systems with 18 ROS particles; (**c**) Density distribution of DOX perpendicular to the cross-sectional plane of carbon nanotubes; (**d**) RDFs of DOX molecules around single-walled carbon nanotubes (inside and outside) at 310 K.

DOX is a polar molecule, and in the presence of an electric field, the dipole moment of DOX is in the same direction as the electric field, causing the molecule to be forced to move in a certain direction. As shown in Figure 4a, the encapsulation behavior under the influence of an electric field alone is first examined, and it is observed that the number of DOX molecules encapsulated by CNTs increases sharply when the electric field reaches 0.35 V/nm. This is attributed to the fact that the electric field induces rapid movement of the polar molecules DOX, facilitating their entry into the CNT interior. Once inside the CNT cavity, electromagnetic shielding is formed, and the stable binding of DOX is ensured by strong van der Waals interactions between DOX and the CNT, greatly enhancing encapsulation efficiency. Here, the number of DOX molecules represents the number of DOX molecules located within the CNT cavity at the end of the simulation, excluding molecules adsorbed on the outer surface or residing near the tube opening. However, under low electric field conditions, DOX tends to aggregate in the solution, forming clusters that resist directional movement, resulting in a low number of DOX molecules entering the CNT. In the system containing 18 ROS particles, the required electric field strength for achieving high encapsulation efficiency is significantly reduced; the number of DOX entering the CNT begins to increase rapidly at an electric field of 0.15 V/nm, and when the field strength reaches 0.3 V/nm, six out of ten DOX successfully enter the CNT. This phenomenon is believed to result from the interaction between ROS and the hydrophilic groups of DOX, which disrupts the π–π stacking between DOX, leading to more DOX molecules being susceptible to movement under the electric field, making them easier to be captured by the CNT. In the system with 18 active particles, as the electric field strength increases, the ability of the CNT to encapsulate and protect DOX is enhanced, reducing the exposure of DOX to ROS and the likelihood of its oxidation. In the system with 24 ROS particles, the encapsulation efficiency is more significantly influenced by the ROS concentration; without an electric field, the number of encapsulated DOX molecules reaches 4, and the encapsulation number remains stable until the field strength increases to 0.45 V/nm, indicating a stronger dependence on ROS concentration rather than the electric field. Within the electric field range of 0.25–0.5 V/nm, the amount of DOX encapsulated in the system with 18 ROS particles is mostly higher than that in the system with 24 ROS particles. It is speculated that in environments with higher ROS concentrations, the frequency of physical collisions between DOX and ROS particles increases significantly. Although this can help suppress DOX aggregation, it may also excessively interfere with DOX mobility, reduce its mean free path, and hinder its directional movement toward the CNT, thereby weakening the encapsulation and protective effects of CNT on DOX. These findings provide important insights for optimizing the strategy of CNT-assisted DOX delivery and protection under the synergistic effects of CAP, particularly in terms of balancing ROS concentration and electric field strength.

To more accurately determine the distribution of DOX, radial distribution functions (RDFs) were calculated with respect to the center of the CNT. The system containing 18 ROS particles under a low electric field of 0.3 V/nm was selected, and the RDF was computed over the last 5 ns of the simulation. It was compared with RDFs obtained under the same field without ROS particles, and in the absence of a field but also with 18 ROS particles. In the RDF analysis, these three systems were chosen as representative systems for mechanistic comparison. As shown in Figure 4d, two distinct RDF peaks are observed: one at approximately 0.8 nm and another at about 1.75 nm. Considering the diameter of the (20,20) CNT is about 1.35 nm, the first peak corresponds to DOX encapsulated inside the CNT, while the second represents DOX adsorbed on the outer surface. Among all conditions, the combination of 0.3 V/nm and active particles produces the highest inner peak, indicating significantly enhanced encapsulation. Although the electric field or active particles alone also contribute to DOX uptake, their combined effect markedly increases encapsulation even at low field strengths. Meanwhile, the ability of CNT to encapsulate and protect DOX is significantly enhanced. As shown in Figure 4c, the density distributions of DOX perpendicular to the cross-sectional plane of carbon nanotubes also support the above finding. In addition, the case with 24 ROS particles at an electric field of 0.3 V/nm was also presented in Figure 4c,d, indicating that a higher ROS concentration does not further improve the encapsulation efficiency under the same electric field, in agreement with the trend shown in Figure 4a. To understand the underlying mechanism, electric fields of different strengths were applied to systems containing 18 ROS particles, and the dipole moment of DOX was analyzed (Figure 4b), showing a rapid increase at the beginning of the simulation with rising field strength, indicating enhanced molecular mobility. Within the 0.1–0.4 V/nm range, the dipole moment decreases after DOX enters the CNT, especially at 0.3 and 0.4 V/nm, suggesting electromagnetic shielding within the CNT. However, at 0.5 V/nm, this reduction slows, and encapsulation efficiency decreases, likely due to high-speed active particles motion under the strong field, which interferes with DOX entry, limiting the protective effect of the CNT. Therefore, optimizing ROS concentration and electric field strength is crucial for efficient CNT-assisted DOX delivery and protection. Within the parameter range investigated here, the combination of 18 ROS particles and an electric field of 0.3 V/nm provides a favorable balance between encapsulation efficiency and relatively mild plasma conditions, whereas 18 ROS particles with an electric field of 0.45 V/nm can achieve higher loading when increased encapsulation is desired. Notably, a further increase in the electric field to 0.5 V/nm does not improve encapsulation efficiency, as discussed above. In addition, increasing the ROS concentration to 24 particles within the same electric field range does not lead to further improvement in encapsulation efficiency. These results suggest that, rather than simply increasing both ROS concentration and electric field strength, a moderate ROS level combined with an appropriate electric field is more favorable for achieving efficient encapsulation. Higher encapsulation reduces the required CNT dosage, minimizing aggregation and associated toxicity, while improving targeting efficiency.

**Figure 5:**
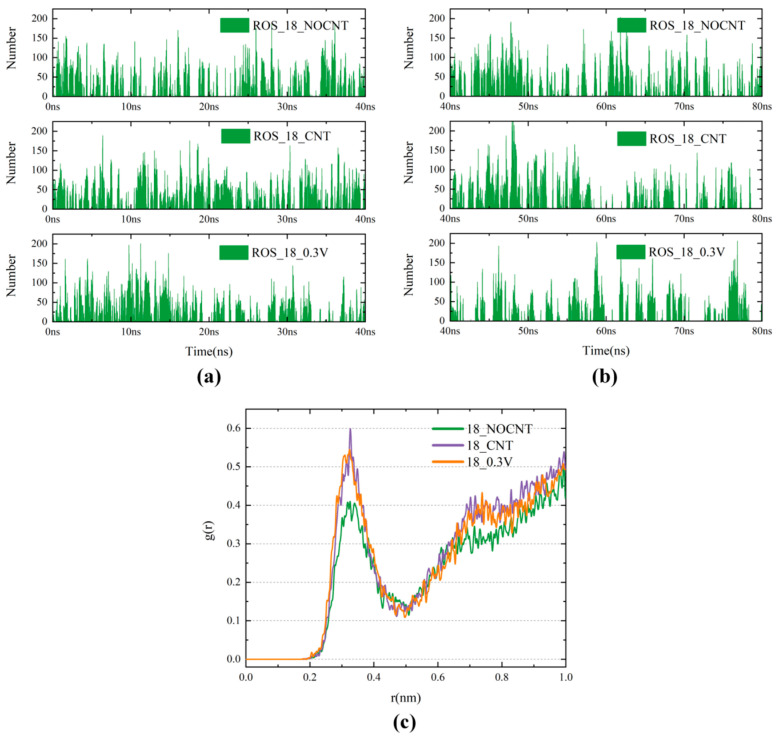
(**a**) Number of contacts between DOX and ROS for the first 40 ns under three conditions: without CNT, with CNT, and with both CNT and an electric field; (**b**) Number of contacts between DOX and ROS for the second 40 ns under three conditions: without CNT, with CNT, and with both CNT and an electric field; (**c**) RDF of ROS around DOX.

To understand at the microscopic level how the presence of CNTs can protect DOX from oxidation when used in combination with CAP, the radial distribution function of ROS around DOX and the contact numbers between DOX and ROS were analyzed, as shown in Figure 5; the RDF shows that ROS is redistributed away from DOX after the addition of CNTs to the system. The analysis of contact numbers in Figure 5a,b also reveals that after the addition of CNT, the number of contacts between DOX and ROS before DOX enters into the CNT shows a slight increase, while the protection of CNT allows the contact number to decrease in the later stage of the 40 ns simulation, and the average contact number increases slightly to 19.74. The introduction of CNT allows DOX to reduce its subsequent contact with ROS following the initial collisions. With both CNT and a 0.3 V/nm electric field applied, the high encapsulation rate results in a significant decrease in the number of contacts after the completion of encapsulation. After extending the simulation time by 40 ns, the average contact number in the system without CNT increases to 17.25 in the last 40 ns, while it decreases to 17.31 with the addition of CNT and 15.48 with the addition of CNT and an electric field, which reflects the protection of DOX by the synergistic effect of CNT and the electric field in a longer time scale, preventing DOX from being oxidized by ROS during combination therapy. Therefore, in the presence of CNTs, the combination of CAP and DOX can be used in the treatment of cancer in a more effective way.

### 3.3. Effect of Plasma Oxidation of POPC Bilayers on Carbon Nanotube Transmembrane Transport

There are two main ways in which CAP destabilizes and disrupts the fluidity of cell membranes. The first is by oxidizing the cell membranes of cancer cells in a way that causes them to collapse more readily, and the second is by the action of an electric field that causes the bilayer to undergo electroporation [49]. Therefore, in this study, we investigated the respective and combined effects of an oxidized phospholipid bilayer (POPC-ALD) and an electric field on CNTs crossing cancer cell membranes. Subsequently, via these effects, we explored how the CAP alters the stability and fluidity of cell membranes and thus affects CNT-assisted drug delivery.

**Figure 6:**
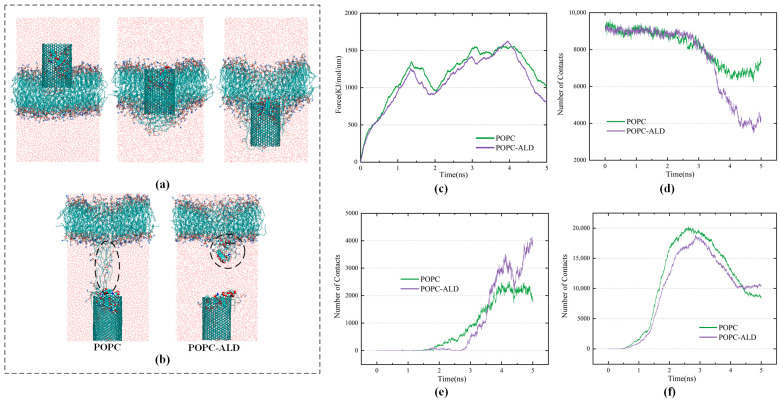
(**a**) Snapshots of the process of carbon nanotubes crossing the POPC model; (**b**) Difference between carbon nanotubes after crossing the pure POPC model and POPC-ALD model; (**c**) Comparison of the pulling force required for carbon nanotubes to cross the POPC model and the POPC-ALD model; (**d**) Contact numbers of carbon nanotubes and DOX; (**e**) Contact numbers of lipid molecules and DOX; (**f**) Contact numbers of CNTs and lipid molecules. In the snapshots, POPC and POPC-ALD are mainly represented by red, blue, and green atoms.

To investigate how the oxidative effect of plasma-generated active particles affects the transport process of CNTs across membranes, two models, namely, a pure phospholipid bilayer and an oxidized phospholipid bilayer, were constructed. The CNT was placed approximately 1.5 nm above the phospholipid bilayer. Figure 6c shows the change in the pulling force of the CNTs during passage through the pure POPC membrane and the oxidized membrane. When the 4-nm-long CNTs are close to the hydrophilic headgroup region of the phospholipid bilayer, the hydrophilic head of the phospholipid bends, and the tensile force increases continuously. This can be attributed to the polarity mismatch between the hydrophobic CNT surface and the hydrophilic environment, which results in an energetic penalty due to the unfavorable exposure of the hydrophobic CNT surface to a polar, hydrophilic environment [50,51,52]. As a result, the forward movement of the CNT is temporarily hindered, leading to a gradual increase in the pulling force. Afterward, as the body of the CNT enters the hydrophobic phase of the bilayer, the local environment becomes more compatible with the hydrophobic nature of the CNT, and the resistance to the movement of the CNT decreases. When the position of the CNT leaves the hydrophobic lipid tail and approaches the hydrophilic lipid headgroup in the lower half of the layer, the applied force increases again, and some lipid molecules extend to the tail end of the CNT, preventing it from leaving the bilayer and exerting a new resistance to the CNT. Finally, as the CNT leaves the bilayer, some of the extracted lipids leave the lipid bilayer with the CNT.

As shown in Figure 6c, under the effect of plasma oxidation, the time taken by the CNT to cross the membrane is shorter, and the pulling force required to cross the cell membrane appears to decrease, with the first peak of the transmembrane decreasing from 1344 kJ/mol/nm to 1258 kJ/mol/nm. This finding suggests that the rigidity of the membrane surface is weakened via oxidation by plasma. The force preventing the CNT from leaving the phospholipid bilayer also decreases after 4000 ps. The analysis in Figure 6b reveals that, under the effect of plasma oxidation, the connection between the phospholipid molecules attached to the CNT and the main body of the phospholipid bilayer is reduced. This reflects the weakening of the mobility and stability of the phospholipid bilayer, which leads to a decrease in the pulling force of the CNT as it leaves the bilayer. In the oxidized membrane model, more lipid molecules enter the interior of the CNT and thus leave the phospholipid bilayer. Notably, the vast majority of them are POPC-ALD, indicating that oxidized lipids are more likely to leave the bilayer with the CNT, which crowds out the space where DOX binds to the CNT. This causes two DOX molecules to leave the interior of the CNT at 3200 ps. Thus, in Figure 6d, the number of contacts between the CNT and DOX in the POPC-ALD model rapidly decreases after 3200 ps, whereas the contact number between the CNT and lipid molecules in Figure 6f increases after the CNT leaves the oxidized bilayer at 4000 ps. Moreover, in Figure 6e, the release of DOX molecules in the POPC-ALD model leads to a rapid increase in the number of contacts between DOX and lipid molecules after 3200 ps, exceeding those in the POPC model. These findings suggest that the oxidation of membrane lipids by plasma accelerates the internalization of CNTs and, at the same time, is more conducive to the release of DOX in target cells.

### 3.4. Effects of Electric Fields on Carbon Nanotube Transmembrane Transport

The length-to-diameter ratio $\frac{L}{D}$ of CNTs, which can reach thousands, can strongly enhance the electric field strength at their ends, and the field enhancement at the tips of CNTs can be estimated by Equation (3):

(3) $$\frac{E}{E_{0}}=\alpha \frac{L}{D}$$

**Figure 7:**
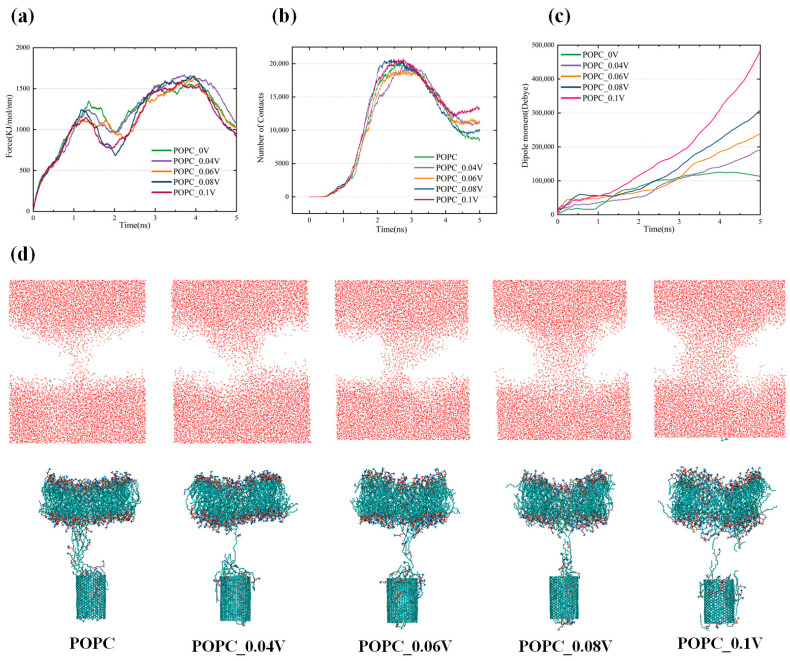
(**a**) Variation in the pulling force required for carbon nanotubes to cross a pure POPC membrane with increasing electric field strength; (**b**) Contact number of CNTs and lipids during carbon nanotube transmembrane crossing; (**c**) The dipole moment of POPC under different electric fields; (**d**) The structure of the water channel of the membrane pore and the bilayer after CNT passed through the pure POPC membrane under electric field.

Therefore, investigating the effects of the electric field on the transmembrane behavior of CNTs is highly important. An electric field of 0.04–0.1 V/nm was applied to the CNT across the pure POPC membrane. As shown in Figure 7a,c, when the electric field strength increases, the dipole moment of POPC increases, and the required pulling force across the membrane of the CNT decreases, which facilitates the transmembrane transport of CNTs. Specifically, when the electric field increases to 0.1 V/nm, the first peak value of the pulling force decreases from 1344 kJ/mol/nm to 1140 kJ/mol/nm. This reduction is 2.37 times that of the pulling force decrease observed when crossing the oxide membrane, and the very small value of the transmembrane force near 2000 ps decreases from 959 kJ/mol/nm to 768 kJ/mol/nm. This suggests that the imposition of the electric field increases the susceptibility of the CNT to internalization by the cell membrane, making the CNT more likely to be transported across the cell membrane. The largest decrease in pulling force is observed between 1000 and 3000 ps. This result reveals that the hydrophobic environment inside the bilayer and the hydrocarbon tails that break off under the action of the plasma significantly decrease the force with the CNT under the action of the electric field. Figure 7d also shows that the number of phospholipid molecules between the CNT and the bilayer after the transmembrane decreases with increasing electric field, which reduces the resistance of the CNT to leave the bilayer.

As shown in Figure 7d, the morphological changes in the bilayer after the CNT crosses a pure POPC membrane under the effects of an electric field are investigated. When the electric field strength is 0, after the CNT passes through the bilayer, the fluidity of the membrane is not damaged. The phospholipid molecules migrate toward the membrane pores generated by the transmembrane, and the membrane pores almost disappear after the CNT leaves the bilayer. This mitigates the damage to the cell membrane caused by the transmembrane. With increasing electric field strength, the number of lipid molecules attached to the outer wall of the CNT leaving the bilayer increases. In addition, the number of membrane pores generated after transmembrane crossing of the CNT increases accordingly. Figure 7b also shows that the number of contacts between the CNT and the lipid molecules increases after the CNT leaves the bilayer. At this time, it is difficult to fill the pores after transmembrane crossing by virtue of the mobility of the bilayer, and the curvature of the membrane also increases, as shown in Figure 7d. When the electric field strength increases to 0.1 V/nm, the radius of the membrane pores is already larger than that of the CNT. This is conducive to the entry of hydrophilic active particles into the interior of the cell along the water channel and can further oxidize key components such as DNA proteins in cancer cells and induce their apoptosis. The electric field intensity that causes severe deformation of the phospholipid membrane in this study is much lower than the threshold that causes electroporation of the bilayer in the study of Wang et al. [49]. This threshold, which causes severe deformation of the phospholipid membrane, is favorable for the transport of DOX into the targeted cancer cells by CNTs, while CAP-generated active particles further enhance the anticancer effect, enabling the inactivation of cancer cells with less damage to healthy cells. Notably, the number of lipid molecules that enter the interior of the CNT under the action of the electric field alone does not increase significantly but rather are attached to the outer wall of the CNT. Thus, the binding of the CNT to DOX does not decrease significantly after the transmembrane, and therefore, synergism between the electric field of the plasma and the oxidative effect is considered.

### 3.5. Synergistic Effects of an Electric Field and Oxidation on the CNTs Passing Through a Membrane

**Figure 8:**
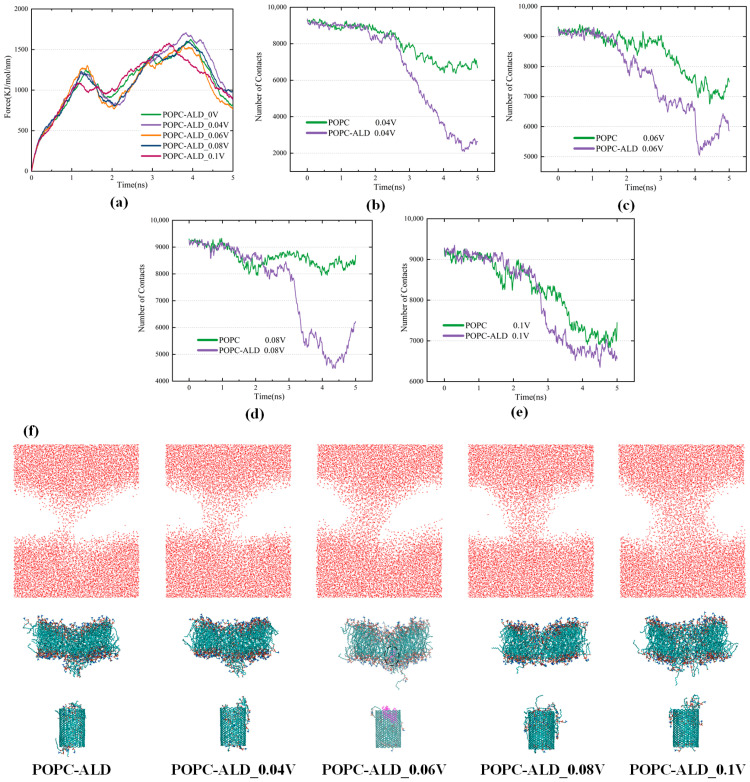
(**a**) Variation in the pulling force required for carbon nanotubes to cross the POPC-ALD membrane with electric field strength; (**b**) Number of contacts of CNTs and DOX during carbon nanotube transmembrane crossing at a 0.04 V/nm field strength; (**c**) Number of contacts of CNTs and DOX during carbon nanotube transmembrane crossing at a 0.06 V/nm field strength; (**d**) Number of contacts of CNTs and DOX during carbon nanotube transmembrane crossing at 0.08 V/nm field strength; (**e**) Number of contacts of CNTs and DOX during carbon nanotube transmembrane crossing at 0.1 V/nm field strength; (**f**) Side views of the system after synergistic transmembrane interaction between the electric field and oxidation effects. In Figure 8f, an adjusted molecular representation and color scheme are used for the POPC-ALD_0.06V snapshot to improve the visibility of the released DOX molecules.

An electric field of 0.04–0.1 V/nm was applied to the CNT crossing the POPC-ALD oxide membrane. The results show that the CNT exhibits excellent transmembrane and drug-carrying properties under the synergistic effects of an electric field and oxidation. It should be noted that the term “synergistic effects” is used here in a qualitative sense to describe the enhanced effect observed when oxidation and the electric field act together, based on comparative simulation results, rather than a strictly defined quantitative measure of synergy. When an electric field of 0.04 V/nm is applied, the first minimal value of the pulling force decreases to 814 kJ/mol/nm, as shown in Figure 8a. Although the decrease in the pulling force is not obvious compared with that at a higher electric field, the interaction between lipid molecules and DOX is stronger at a relatively lower electric field, and the binding force of DOX to CNTs becomes poorer (as shown in Figure 8b–e). This facilitates drug release, ultimately resulting in the delivery of DOX molecules into the intracellular environment. When the electric field reaches 0.06 V/nm, the pulling force further decreases, and the first minimum of the pulling force drops to the lowest value of 790 kJ/mol/nm. The release of DOX molecules can also be identified in Figure 8f, where the molecular display mode and color scheme were adjusted to improve the visibility of the released DOX molecules. The enhanced internalization of CNTs by the cell membrane and the enhanced ability to release DOX within the targeted cells suggest that this is the optimal electric field strength for facilitating CNT translocation across the cancer cell membrane under the synergistic effect of the electric field and oxidation. This optimal electric field strength achieves a good balance between CNT transmembrane transport and intracellular DOX release. When the electric field is increased to 0.1 V/nm, the transmembrane behavior of the CNT changes significantly, with the first peak of the pulling force decreasing to only 1076 kJ/mol/nm. In addition, the speed of transmembrane crossing further accelerates, reaching the second peak at 3400 ps. This is approximately 600 ps earlier than that of the low electric field. Moreover, the pulling force to leave the bilayer is also significantly reduced. However, when the field strength is too high, the contact between lipid molecules and DOX decreases, and the binding of DOX to CNTs is greater, resulting in a relatively high number of contacts between DOX and CNTs after transmembrane crossing (as shown in Figure 8b–e), thereby inhibiting drug release. Remarkably, the contact number of DOX with the CNT remains stable until 2500 ps, when the CNT leaves the bilayer and enters the intracellular space, thereby preventing premature drug release.

Overall, the pulling force required for the transport of CNTs through the transmembrane decreases compared to that when the electric field is applied alone. In addition, the oxidation of the cell membrane reduces the number of lipids connecting the CNT to the main body of the phospholipid bilayer, and the force preventing the CNT from exiting the bilayer decreases. These factors consequently promote CNT transmembrane transport. The POPC-ALD entering the interior of the CNT increases, squeezing the space for DOX to bind with the CNT, resulting in the release of DOX more readily in the target cells. The membrane pores after the CNT transmembrane are also enlarged, which favor the entry of active particles. Compared with oxidation alone, the pulling force of CNTs decreases significantly after entering the interior of the bilayer, and the first minimum of the pulling force drops to the lowest value of 790 kJ/mol/nm at an electric field of 0.06 V/nm. This is presumed to be related to the behavior of the negatively charged oxygen atoms carried by the tails at the hydrophobic end of the alkane chain that are broken off by the action of the plasma in the presence of an electric field. The synergistic combination of an electric field and the oxidative effect of the plasma reveals the advantages of a plasma-assisted drug delivery system.

## 4. Conclusions

In this study, the classical molecular dynamics methods were employed to simulate the adsorption and encapsulation of doxorubicin (DOX) by carbon nanotubes (CNTs) during the combined treatment of plasma and DOX, as well as the process of drug-loaded CNTs passing through the plasma-oxidized POPC cell membrane. The results of the adsorption and encapsulation study indicate that the presence of CNTs enables DOX to efficiently enter the inner cavity of CNTs in the presence of medium to high concentrations of active particles, thus preventing the drug from being oxidized during the combined treatment with cold atmospheric plasma (CAP). Under the combined effect of the electric field and active particles, the directional movement of the polar molecule DOX makes it more likely to be captured by CNTs, thereby reducing their exposure to reactive oxygen species (ROS) and the likelihood of oxidation. In the presence of an electric field of 0.3 V/nm and 18 ROS particles, the encapsulation rate reaches 60%, which improves the therapeutic efficiency of DOX and provides new insights for the combined treatment of cancer using plasma and chemotherapeutic drugs.

In terms of the ability of drug-loaded CNTs to cross lipid membranes under plasma effects, the oxidative effect of CAP together with the electric field enhances the intracellular internalization of the CNTs and facilitates their entry into the intracellular space. The oxidative effect reduces the rigidity of the membrane, and the oxidized lipid molecules also make it easier for CNTs to release DOX after entering the intracellular space. The synergistic effect of the oxidation effect and the electric field further improves the cell membrane penetration ability of the CNTs. The first peak of the transmembrane decreases from 1344 kJ/mol/nm to a minimum of 1076 kJ/mol/nm. Meanwhile, the number of membrane pores generated by the transmembrane increases, facilitating the entry of active particles into the cell to oxidize key components of cancer cells. This shows the potential of the CNT drug delivery system for targeting cancer cells under the synergistic effect of plasma components. The synergistic effect of CAP and the CNT drug delivery system not only enhances drug delivery efficiency but also avoids oxidation of the drug during administration, which is expected to provide a more precise therapeutic strategy for cancer treatment.

Although molecular dynamics simulation studies provide valuable insights, there are still discrepancies between the simulated conditions and an actual biological environment. In future studies, an animal cell membrane model will be used, and the simulation parameters will be optimized to further validate the effect of plasma on the transmembrane process of CNTs, aiming to achieve greater biological relevance and accuracy. We also plan to use reactive molecular dynamics simulations to study the oxidative effects of CAP on drug-loaded carbon nanotubes. From both classical and reactive molecular dynamics perspectives, we aim to elucidate the potential of CNTs in the synergistic interaction between CAP and anticancer drugs. With subsequent experimental validation and preclinical studies, this strategy is expected to provide more precise and efficient therapeutic options with lower toxicity and side effects for cancer chemotherapy.

## Data Availability

The data in this study are available on request from the corresponding author.
